# Osteosynthesis using Scorpion plate for neer type V distal clavicle fracture

**DOI:** 10.1186/s12891-024-08039-z

**Published:** 2024-11-14

**Authors:** Ryogo Furuhata, Noboru Matsumura, Yusaku Kamata, Atsushi Tanji

**Affiliations:** 1 Department of Orthopaedic Surgery, Ashikaga Red Cross Hospital, 284-1 Yobe-cho, Ashikaga-shi, 326-0843 Tochigi Japan; 2 Department of Orthopaedic Surgery, Keio University School of Medicine, Shinjuku-ku, Tokyo, Japan; 3 Department of Orthopaedic Surgery, National Hospital Organization Tokyo Medical Center, Meguro-ku, Tokyo, Japan

**Keywords:** Distal clavicle fracture, Plate fixation, Outcome, Complication, Osteosynthesis, Scorpion plate

## Abstract

**Background:**

Neer type V distal clavicle fractures are considered the most unstable fracture type and are characterized by the disruption of continuity between the coracoclavicular (C-C) ligaments and proximal or distal bone fragments. However, owing to the rarity of such fractures, there is currently no universally accepted surgical procedure for their treatment. Recently, the scorpion plate, an anatomical, non-locking, pre-contoured plate with two grasping arms to fix the distal or inferior clavicular fragments, was introduced. This study aimed to investigate the postoperative functional and radiological outcomes of osteosynthesis using only scorpion plates in Neer type V fractures.

**Methods:**

We retrospectively identified 23 patients who underwent scorpion plate fixation for Neer type V fractures at two general hospitals. All patients underwent only plate fixation without C-C ligament augmentation. Subsequently, we investigated their postoperative functional outcomes, complication rates, and modified C-C distance ratio at 1 year.

**Results:**

The mean postoperative Constant score was 96 ± 5, with all cases achieving bone union. Complications within 1 year postoperatively included plate loosening in one patient (4.3%) and plate irritation in two patients (8.7%). Additionally, the modified C-C distance ratio averaged 114 ± 15%.

**Conclusions:**

This study offers novel insights into the management of Neer type V distal clavicle fractures. Our findings indicate that osteosynthesis using only scorpion plates can lead to satisfactory functional outcomes with minimal complications.

## Background

Unstable distal clavicle fractures, including those classified as Neer’s types II and V [1, 2], often exhibit a high nonunion rate with conservative treatment [3, 4], prompting consideration for surgical intervention [5]. Particularly, the treatment of Neer type V fractures poses considerable challenges because of the disruption of continuity between the coracoclavicular (C-C) ligaments and proximal or distal bone fragments, leading to increased instability [6]. Although plate fixation stands as a well-established surgical procedure for managing unstable distal clavicle fractures, yielding generally favorable postoperative outcomes [7–10], clinical studies have indicated that plate fixation for Neer type V fractures is associated with a higher incidence of postoperative complications, stiffness [11], and more severe postoperative C-C separation [12] compared to that for type II fractures. Consequently, treating Neer type V distal clavicle fractures presents considerable difficulties for orthopedic surgeons. However, owing to the rarity of Neer type V fractures [13], there is limited research available on treatments specific to this fracture type [6, 14].

**Fig. 1:**
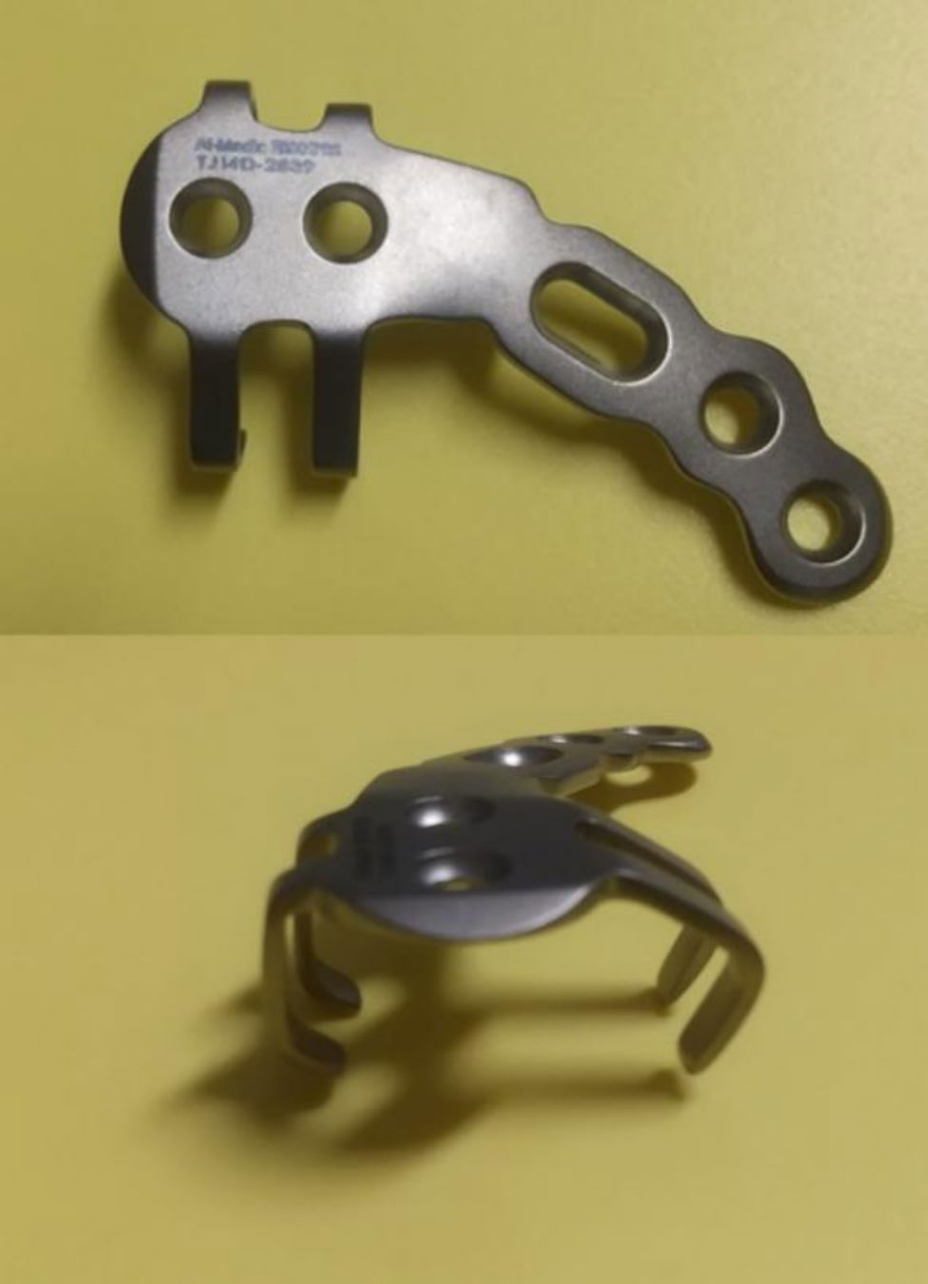
Photographic images of the scorpion plate

Recently, the scorpion plate, an anatomical non-locking pre-contoured plate characterized by two grasping arms designed to stabilize the distal or inferior clavicular fragments, along with screws, has been introduced [15] (Fig. 1). A biomechanical study reported that scorpion plate fixation had torsional and bending stiffness significantly higher than that of tension band wiring and equivalent to that of hook plate fixation [16]. Furthermore, a satisfactory bone union rate was demonstrated for Neer type II and V distal clavicle fractures treated only with a scorpion plate [15]. However, the postoperative functional outcomes of scorpion plate fixation on the shoulder have not been thoroughly investigated; as such, the surgical outcomes of scorpion plate fixation for Neer type V fractures remain unknown.

This study aimed to investigate the postoperative functional and radiological outcomes of osteosynthesis using only scorpion plates in Neer type V distal clavicle fractures.

## Methods

### Study design and patients

The study protocol was approved by the independent ethics committee of our hospital. We retrospectively investigated patients who underwent osteosynthesis using the SCORPION^®^ NEO plate (Aimedic MMT, Tokyo, Japan) for Neer type V distal clavicle fractures at two general hospitals from 2017 to 2019 and 2022 to 2023 (excluding the coronavirus disease 2019 pandemic period). During this timeframe, all distal clavicle fractures were exclusively managed using a scorpion plate without C-C ligament augmentation for osteosynthesis. Inclusion criteria comprised acute traumatic fractures operated on within 3 weeks of injury and adult patients with closed epiphysis. Exclusion criteria included failure to adhere to follow-up for at least 1 year after surgery and paralysis of the affected upper limb owing to cerebral infarction or other causes.

### Surgical procedure

**Fig. 2:**
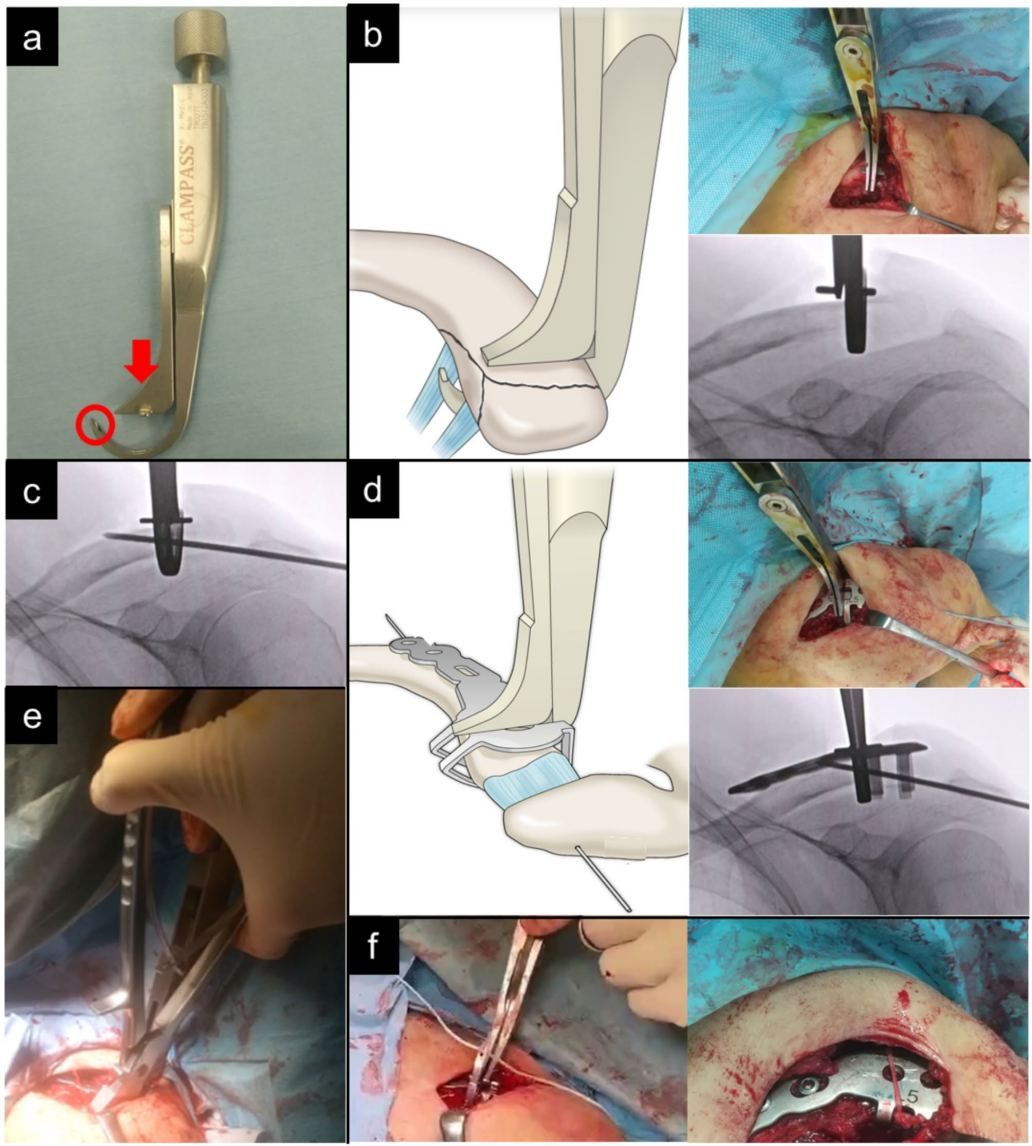
Clinical photographs, radiographs, and a schematic representation of scorpion plate fixation for Neer type V distal clavicle fracture. (**a**) An image of CLAMPASS^®^, an instrument for fracture reduction. This tool allows for the reduction of bone fragments by applying pressure in the direction of the red arrow. This tool also has a suture hole for suturing the plate and clavicle (red circle). (**b**) Proximal and inferior bone fragments were crimped using the CLAMPASS^®^, and (**c**) a Kirschner wire was inserted from the lateral side of the acromion for temporary fixation. (**d**) Following the application of the SCORPION^®^ NEO plate, the plate and bone fragments were crimped using CLAMPASS^®^. (**e**) After screw insertion, the plate arms were bent using a specialized bender. (**f**) A high-strength non-absorbable suture was passed through the suture hole of the CLAMPASS^®^, and the clavicle and plate were sutured circumferentially

**Fig. 3:**
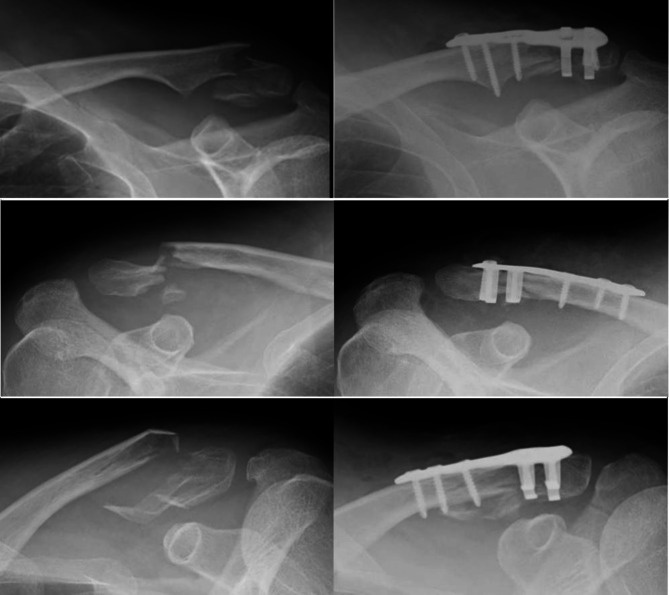
Radiographs illustrating scorpion plate fixation for Neer type V distal clavicle fractures. The left panel displays preoperative radiographs, while the right panel presents postoperative radiographs

All surgeries were conducted with patients positioned in the beach chair posture under general anesthesia. Incisions were made along the long axis above the clavicle. Fracture fragments were reduced using the CLAMPASS^®^, an instrument designed for reduction (Fig. 2a). Following the passage of the lower end of the CLAMPASS under the inferior fragment, proximal and inferior bone fragments were compressed (Fig. 2b). Subsequently, a 2.0-mm Kirschner wire was temporarily inserted from the lateral side of the acromion to secure the reduction (Fig. 2c). We determined the position of the plate placement such that the proximal plate arm could grasp the inferior fragment and the distal plate arm could grasp the inferior cortex of the distal fragment under direct vision and fluoroscopy. In particular, we prioritized being able to securely hold the inferior fragment, because surgery for Neer type V fractures is crucial for stabilizing the inferior fragment with attached C-C ligaments [17]. After placing the SCORPION^®^ NEO plate supraclavicularly, the plate and bone fragments were further compressed using the CLAMPASS^®^ (Fig. 2d). Three cortical screws were inserted into the proximal screw holes, with zero to two screws placed in the distal screw holes. A specialized bender was employed to shape the two arms of the plate (Fig. 2e). Notably, the plate arm was bent to ensure that the inferior bone fragments were captured, with confirmation via radiography. Subsequently, a high-strength non-absorbable suture was passed through suture hole of the CLAMPASS^®^, and the clavicle and plate were sutured circumferentially to reinforce the fixation of the inferior bone fragment and plate (Fig. 2f). Postoperative radiographs are shown in Fig. 3.

Post-surgery, the affected arm was immobilized with a sling for 1–2 weeks. Active range of motion exercises and rotator cuff exercises with scapular fixed were initiated after 1 week post-surgery. Full active range of motion was permitted 3 weeks post-surgery, and scapular training exercises were started 2 months post-surgery. Patients were permitted to resume heavy labor activities 3 months post-surgery and to resume sports activities 6 months post-surgery. Implant removal was conducted once the bone union was achieved at least 6 months post-operation and upon patient request.

### Outcome measures

We evaluated the postoperative shoulder functional outcomes using the Constant score [18] and assessed postoperative shoulder pain using the visual analog scale (VAS) 1 year postoperatively. Additionally, postoperative complications (such as nonunion, plate loosening, plate irritation, and stiffness) occurring within 1 year post-surgery were also examined. A single examiner evaluated these outcomes from medical records and plain radiographs. As previously described [19], nonunion was defined as the absence of bone bridging for > 12 months post-surgery. Plate irritation was assessed based on patient complaints recorded immediately before plate removal, and postoperative C-C separation was evaluated using a modified C-C distance ratio. We defined the modified C-C distance as the vertical distance between the upper border of the coracoid process and the upper border of the clavicle on plain radiographic images of both clavicles obtained simultaneously in the standing position [14]. The modified C-C distance ratio was calculated by comparing the modified C-C distance with that of the unaffected side, expressed in percentage.

### Statistical analysis

All statistical analyses were conducted using the SPSS software (version 27.0*, IBM, Armonk, NY, USA). Continuous data are expressed as mean ± standard deviation, and categorical data are presented as numbers and percentages.

## Results

We identified a total of 26 patients who met our inclusion criteria. Among them, two patients were excluded because of loss to follow-up, and one was excluded owing to upper limb paralysis resulting from cerebral hemorrhage. Table 1 presents the demographic characteristics of the patients. The average age at the time of surgery was 54 ± 19 years (range: 16–89 years). All surgeries were performed by three surgeons with > 10 years of experience in trauma surgery. The mean operative time was 81 ± 24 min, and the mean blood loss was 39 ± 53 g.

**Table 1:** Patient characteristics

	Value (*N* = 23)
Age * (years)	54 ± 11
Sex †(Female/ Male)	4/ 19
Affected arm †(Dominant/ Non-dominant)	14/ 9
Smoking †	10
Diabetes †	3
Time from injury to surgery * (days)	6 ± 4

*Values are presented as mean ± standard deviation. †Values are presented as the number of patients.

At 1 year postoperatively, the mean Constant score was 96 ± 5 (range: 80–100), and the mean VAS was 0.5 ± 0.9 cm (range: 0–3.0 cm). The average range of shoulder motion for anterior elevation was 157 ± 16° (range: 120–170°), and that for external rotation was 49 ± 13° (range, 20–60°). Among complications observed within 1 year postoperation, nonunion was not observed (0%), plate loosening occurred in one patient (4.3%), plate irritation in two patients (8.7%), and no patient experienced stiffness (0%). For the one patient who developed postoperative plate loosening, we did not perform any additional surgery or treatment because satisfactory bone union and functional outcome were obtained. However, for the two patients who developed plate irritation, we removed the implant after bone union. The mean modified C-C distance ratio at 1 year postoperatively was 114 ± 15% (range: 100–146%) (Table 2).

**Table 2:** Clinical outcomes of Scorpion plate fixation at 1-year postoperatively

Outcome	Value (*N* = 23)
Constant score *	96 ± 5
Nonunion †	0
Plate loosening †	1
Plate irritation †	2
Stiffness †	0
Modified coracoclavicular distance ratio * (%)	114 ± 15

*Values are presented as mean ± standard deviation. †Values are presented as the number of patients.

## Discussion

This study investigated the postoperative outcomes of employing scorpion plates for the osteosynthesis of Neer type V distal clavicle fractures. Our results indicate that scorpion plate fixation resulted in a satisfactory bone union rate and functional outcome, with few complications observed.

To date, only two studies have specifically examined postoperative outcomes related to Neer type V distal clavicle fractures: one involving osteosynthesis with a hook plate [14] and the other utilizing an anatomical locking plate with additional C-C ligament augmentation [6]. The mean Constant score obtained in this study (96 ± 5) was comparable to the previously reported functional outcomes [6, 14]. Similarly, postoperative shoulder pain and shoulder motion observed in this study aligned with those from previously reported studies [6, 14]. However, the overall complication rate associated with hook plate fixation for Neer type V fractures was reported to be as high as 45%, encompassing complications such as postoperative stiffness, subacromial erosion, posttraumatic acromioclavicular joint arthrosis, and peri-implant fractures [14]. In contrast, complications such as plate irritation and plate loosening occurred in only 8.7% and 4.3% of our cases, respectively, indicating that the complication rate of scorpion plate fixation was lower than that of hook plate fixation. Regarding the postoperative modified C-C distance ratio, we observed a ratio of 114%, which was slightly higher than that reported for hook plate fixation [14] or plate fixation with C-C ligament augmentation [6]. However, research has shown that an increased C-C distance postoperatively does not significantly affect shoulder function or bone union [20, 21], which is consistent with our findings.

Surgery for Neer type V fractures is crucial for stabilizing the inferior bone fragment with attached C-C ligaments, alongside fixation of the proximal and distal fragments [17]. However, achieving secure fixation with pre-contoured plate fixation alone can be challenging [6]. We hypothesized that the scorpion plate design addresses this challenge by enabling the two plate arms to grasp and stabilize the inferior bone fragment, potentially contributing to the satisfactory functional outcomes and bone union rates observed in this study. Suturing of plates and bone fragments using the suture hole in CLAMPASS is also a procedure used to reinforce the fixation of the plate and inferior bone fragment in Neer type V fractures. However, a previous study reported that this suture procedure had no significant effects on the frequency of postoperative complications and postoperative C–C distance ratios in Neer type II and V fractures [15]; therefore, its clinical significance remains unclear.

Osteosynthesis using a scorpion plate presents two advantages over other plates and C-C ligament augmentation techniques: (i) it allows for the fixation of the distal and inferior bone fragments with two plate arms, thereby preventing fragmentation during screw insertion or bone-hole creation, and (ii) it eliminates the risk of complications associated with C-C ligament augmentation, such as anchor or button pull-out, coracoid fracture, or brachial plexus injury [17, 22–24]. However, it is worth noting that the absence of locking screws in the scorpion plate design may increase the risk of plate loosening.

The results of our study and that of a previous study [15] suggest that osteosynthesis using the scorpion plate alone is applicable to Neer type IIA, IIB, and V distal clavicle fractures. However, the type IIC fracture reported by Cho et al. [25], wherein the conoid and rhomboid ligaments are torn, is not indicated, because severe C-C instability remains after only plate fixation. In addition, the scorpion plate has only three screw holes for the proximal fragment and does not have a longer plate type; therefore, it is not suitable for types of fractures where the fracture line is long and extends to the proximal screw hole.

This study had three major limitations. First, this was a nonrandomized retrospective study without a control group; thus, the surgical outcomes of scorpion plates could not be compared with those of other surgical procedures. Second, despite Neer type V fractures accounting for only 9% of all distal clavicle fractures [13], the sample size employed in this study was relatively small. Third, although functional and radiological outcomes were evaluated at 1 year postoperatively, long-term outcomes were not assessed.

## Conclusion

This study offers valuable insights into the treatment of Neer type V distal clavicle fractures. Our findings suggest that osteosynthesis using only scorpion plates can achieve satisfactory functional outcomes with minimal complication rates.

## Data Availability

Data supporting this study’s findings are available from the corresponding author on reasonable request.

## Abbreviations

C-C: Coracoclavicular
VAS: Visual analog scale
